# Authentication of *Citrus* spp. Cold-Pressed Essential Oils by Their Oxygenated Heterocyclic Components

**DOI:** 10.3390/molecules27196277

**Published:** 2022-09-23

**Authors:** Noura S. Dosoky, Prabodh Satyal, William N. Setzer

**Affiliations:** 1 Aromatic Plant Research Center, Lehi, UT 84043, USA; 2 Department of Chemistry, University of Alabama in Huntsville, Huntsville, AL 35899, USA

**Keywords:** essential oils, *Citrus*, furanocoumarins, coumarins, UPLC-MS/MS

## Abstract

*Citrus* essential oils are routinely adulterated because of the lack of regulations or reliable authentication methods. Unfortunately, the relatively simple chemical makeup and the tremendous price variations among *Citrus* varieties encouraged the interspecies adulteration of citrus oils. In this study, a sensitive UPLC-MS/MS method for the quantitation of 14 coumarins and furanocoumarins is developed and validated. This method was applied to screen the essential oils of 12 different *Citrus* species. This study, to our knowledge, represents the most comprehensive investigation of coumarin and furanocoumarin profiles across commercial-scale *Citrus* oils to date. Results show that the lowest amount was detected in calamansi oil. Expressed oil of Italian bergamot showed the highest furanocoumarin content and the highest level of any individual furanocoumarin (bergamottin). Notable differences were observed in the coumarin and furanocoumarin levels among oils of different crop varieties and origins within the same species. Potential correlations were observed between bergapten and xanthotoxin which matches with known biosynthetic pathways. We found patterns in furanocoumarin profiles that line up with known variations among the *Citrus* ancestral taxa. However, contrary to the literature, we also detected xanthotoxin in sweet orange and members of the mandarin taxon. Using multivariate analysis, we were able to divide the *Citrus* oils into 5 main groups and correlate them to the coumarin compositions.

## 1. Introduction

*Citrus* essential oils (EOs) have several applications in cosmetics, the food industry, and the flavor and fragrance industry. They are also utilized as natural preservatives because of their wide range of biological activities, which include antioxidant and antimicrobial actions [1]. These strong biological activities are attributed to the presence of terpenes, flavonoids, carotenes, and coumarins [2]. Several studies have investigated the volatile makeup of various parts of *Citrus* species due to their significant economic importance. All cold-pressed *Citrus* oils contain a portion of non-volatiles fundamentally made of simple coumarins, psoralens, and methoxy-flavones [3]. Coumarins (1,2-benzopyrones) are a huge family of naturally occurring secondary metabolites. Psoralens, also known as furanocoumarins (FCs), are a large family of compounds commonly found in Rutaceae, Apiaceae, and Fabaceae, with Rutaceae containing the highest concentrations [4,5]. FCs contain a furan ring fused to a coumarin core [6]. The fusion helps separate the FCs into linear or angular structural forms. FCs have shown a potential to elicit variable degrees of phototoxic skin reactions. In comparison to angular FCs, linear FCs have often been proven to cause phototoxic responses at lower doses [7].

While studying the volatile composition of *Citrus* oils, the nonvolatile fractions are hard to detect under standard gas chromatography conditions because of their limited volatilities, relatively polar or heat-liable nature. These nonvolatile ingredients may hold the secret to constructing a perfect analytical strategy for interspecies adulteration detection. This essential fraction of the cold-pressed oil can be used to identify species-specific patterns and establish *Citrus* species fingerprinting. For instance, creating synthetic bergamot oils or adulterating bergamot oils with similar *Citrus* oils like bitter orange are simple strategies to boost profits. Both strategies make it essentially impossible for consumers to detect the difference. The non-volatile fraction contributes very little to the *Citrus* oils’ aroma, but because of its high complexity, commercial unavailability, or extremely high cost in comparison to the *Citrus* oils themselves, it is more difficult to manipulate. Previous studies report the separation and identification of coumarins and FCs in *Citrus* peel extracts and oils using gas chromatography–mass spectroscopy (GC-MS) after derivatization [8], high-performance liquid chromatography (HPLC) [8], enzyme-linked immunosorbent assay (ELISA) [9], reversed-phase (RP)-HPLC [10,11], HPLC-diode array detector (DAD) [12], HPLC-nuclear magnetic resonance (NMR) [13], ultra-performance liquid chromatography coupled with mass spectrometry (UPLC-MS) [14,15], LC-MS [16], and HPLC-UV-MS [17].

**Figure 1:**
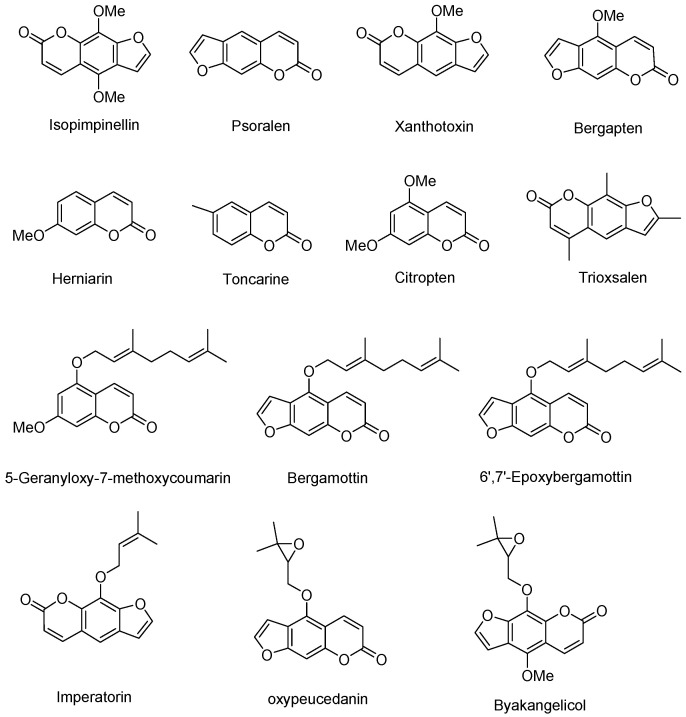
Chemical structure of key non-volatile components in expressed *Citrus* essential oils.

The objective of the present study was to develop a sensitive UPLC-MS/MS method to quantify 14 selected coumarins and FCs (Figure 1). This validated method was then applied to the cold-pressed essential oils of bergamot (*Citrus bergamia* Risso & Poit), bitter orange (*C. aurantium* L.), calamansi (*C.* × *microcarpa* (Bunge) Wijnands), clementine (*C. clementina* Hort. ex Tanaka), grapefruit (*C.* × *paradisi* Macfady), kumquat (*C. japonica* Thunb.), lemon (*C. limon* Osbeck), lime (*C. aurantifolia* (Christm.) Swingle), mandarin (*C. reticulata* Blanco), sweet orange (*C. sinensis* L.), tangerine (*C. tangerina* Hort. ex Tanaka), and yuzu (*C. junos* Sieb. ex Tanaka) as well as petitgrain EO.

## 2. Results and Discussions

### 2.1. Method Validation

**Figure 2:**
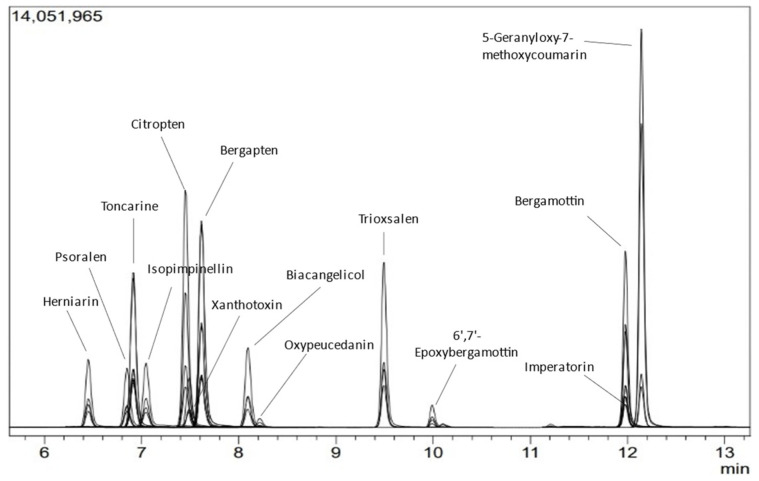
LC-MS/MS chromatogram (MRM acquisition mode) of 14 targeted coumarins using a Shimadzu LCMS8060.

The LC-MS/MS chromatogram of 14 coumarins using the MRM acquisition mode is shown in Figure 2. Specificity, precision, accuracy, linearity, intermediate precision, and LOQ results are summarized in Table 1. The method proved specific to the target compounds since no interferences were found in any of the processed blanks. All compounds met the acceptance criterion of RSD% ≤ 10 based on the precision and intermediate precision results. Compound recovery percentages ranged from 94.07 to 114.53% of the expected value. The linearity of the calibration curve of the 14 compounds was well correlated (r ≥ 0.98) within a range of 0.0001–0.1 ppm. The LOQ values ranged from 0.0001 to 0.005 ppm. These findings demonstrate that the developed method is suitable for analyzing the 14 targeted compounds in EOs.

### 2.2. Comparison of Citrus EO Coumarin and Furanocoumarin Content

*Citrus* EOs used in this study were produced by expression in industrial settings. A total of 374 *Citrus* EOs were screened for coumarins using a 20 min UPLC-MS/MS method targeting 14 coumarins, of which 10 are linear furanocoumarins. The compositions of target compounds greatly differed among the tested *Citrus* EOs (Table 2). The least quantity of coumarins and FCs was detected in calamansi EO (0.15 ± 0.02 ppm). In comparison, the largest presence of coumarins and FCs was found in Italian bergamot EO (171,453.11 ± 9227.11 ppm), followed by the Brazilian bergamot EO (52,473.90 ± 1775.63 ppm). Expressed oil of Italian bergamot showed the highest FC content (167,281.60 ± 1017.74 ppm) and the highest level of any individual FC (109,730.67 ± 3150.55 ppm bergamottin). Notable differences were observed in the coumarin and FC levels among EOs of different crop varieties and origins within the same *Citrus* species. There have been several previous investigations on the non-volatile components of *Citrus* essential oils reported in the literature (Table 3). The non-volatile components are far more species-specific than the volatile components, which have comparable patterns in different *Citrus* oils. We found patterns in FC profiles that correspond with published differences among the *Citrus* ancestral taxa [15,18]. Our findings are in line with previous reports that found a mixture of FCs from the bergapten, xanthotoxin, and isopimpinellin clusters in EOs derived from the citron (*C. medica*) and papeda (*C. micrantha*) ancestral taxa [15]. In this study, EOs derived from fruits of the mandarin taxa (mandarin, clementine, and tangerine) showed low total coumarin (2.44–149.45 ppm) and FC levels (2.44–149.45 ppm), not aligning with a previous report that this taxon is nearly devoid of FCs [14,15]. Interestingly, trioxsalen and toncarine were not detected in any of the *Citrus* EOs. Epoxybegamottin was absent from calamansi, clementine, mandarin, kaffir lime, and petitgrain oils. The content of psoralen was almost negligible in most of the *Citrus* EOs but was relatively high in white grapefruit EO (82.65 ± 0.76 ppm). Furthermore, large amounts of xanthotoxin were detected in bergamot and lime EOs. Previous reports indicate that xanthotoxin is absent from sweet orange (*C. sinensis*, pummelo taxon) and the mandarin taxa [14,15] while we found 4.85 ± 0.32 ppm in sweet orange EO and 0.33–15.24 ppm xanthotoxin in the mandarin taxa EOs. Bergamottin and 5-geranyloxy-7-methoxycoumarin were reported in mandarin, lemon, and lime oils but not in orange oil [17]. The differences between our findings and previous studies could be due to genetic and/or environmental impacts on FC biosynthesis [19]. Our LOQ, however, may be lower than that of other reports because it was based on the weight of EO rather than the weight of fresh fruit peel. Alternative explanations for the differences in our findings include genetic admixture in *Citrus* varieties or contamination during processing and handling.

### 2.3. Multivariate Analysis

**Figure 3:**
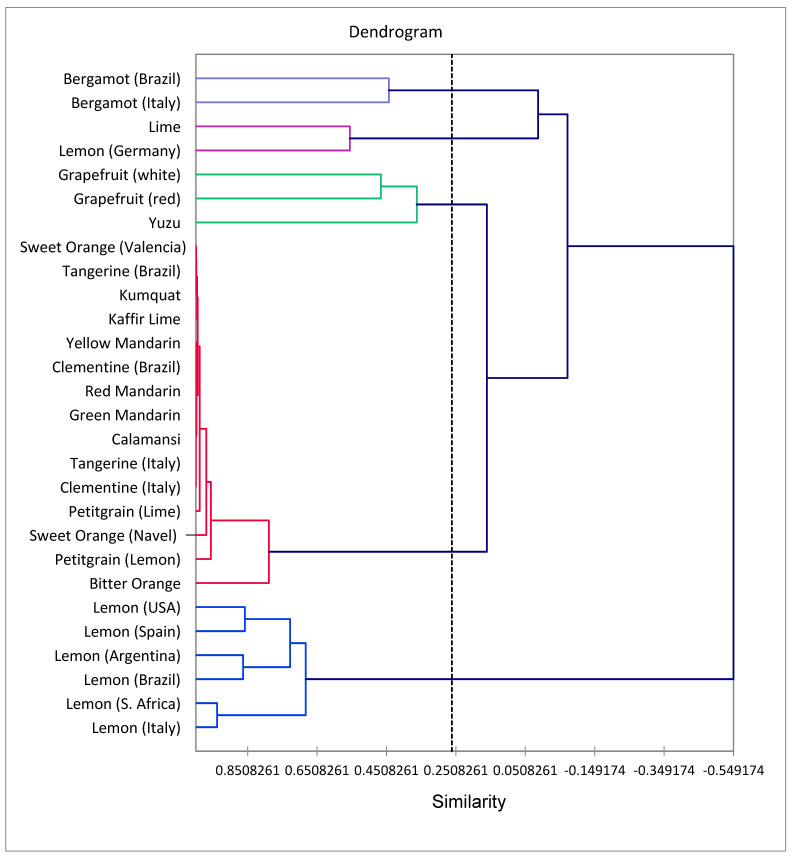
Dendrogram obtained by cluster analysis of the coumarin composition of *Citrus* essential oils, based on correlation and using the unweighted pair-group method with arithmetic average (UPGMA).

**Figure 4:**
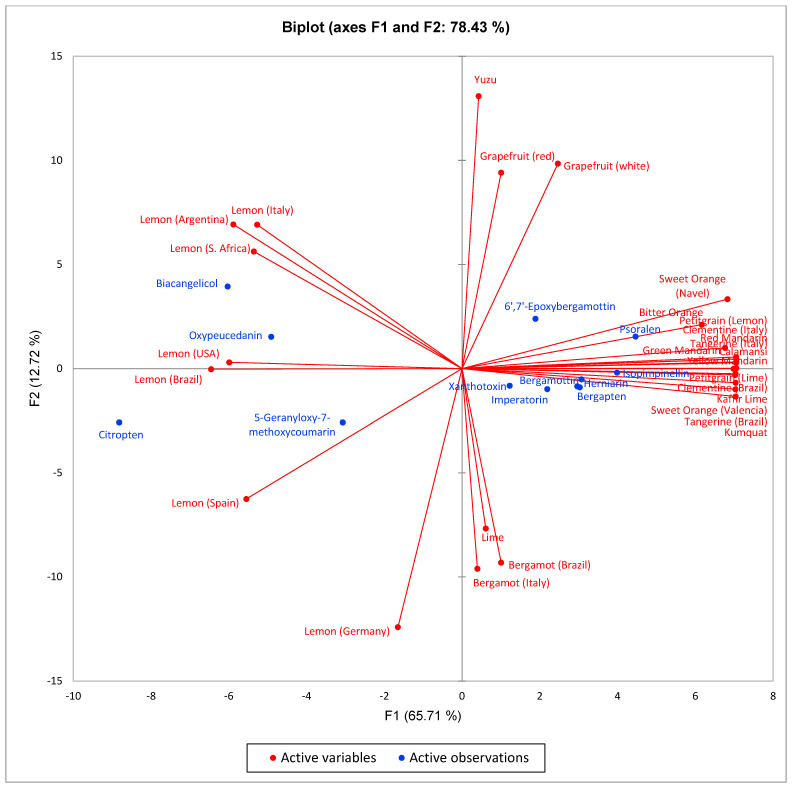
Principal component biplot of PC1 and PC2 scores and loadings indicating the coumarin chemical relationships of *Citrus* essential oils.

In order to examine the similarities and relationships between the coumarin compositions and the *Citrus* essential oils, AHC and PCA were carried out based on a data matrix comprised of 28 *Citrus* “types” and 12 coumarin components. Based on > 25% similarity, the AHC shows five groups (Figure 3): Group 1 (bergamot from Italy and bergamot from Brazil), Group 2 (lime and lemon from Germany), Group 3 (yuzu, red and white grapefruit), Group 4 (a large group composed of oranges, tangerines, clementines, mandarins calamansi, and petitgrains), and Group 5 (lemons). The PCA analysis (Figure 4) of the *Citrus* essential oils indicates that F1 and F2 explain 78.43% of the variation in coumarin compositions among the *Citrus* types. The bergamot group (Group 1) is positively correlated with bergamottin, bergapten, and xanthotoxin; the lemon group (Group 5) positively correlates with biacangelicol and oxypeucedanin as well as citropten and 5-geranyloxy-7-methoxycoumarin. The grapefruit and yuzu group (Group 3) correlate with 6ʹ,7ʹ-epoxybergamottin and psoralen. Group 4 (oranges, mandarins, clementines, etc.) are characterized as having relatively low levels of coumarins. A positive correlation was found between bergapten and xanthotoxin (2 structures related by a common precursor in biosynthesis [15]).

## 3. Materials and Methods

### 3.1. Chemicals

Xanthotoxin, herniarin, toncarine, bergamottin, oxypeucedanin, biacangelicol, psoralen, isopimpinellin, bergapten, and imperatorin (purity ≥ 98%) were purchased from Chengdu Alfa Biotechnology (Chengdu, China). 5-Geranyloxy-7-methoxycoumarin (purity ≥ 99%) was bought from Extrasynthese (Genay, France). Trioxsalen and 6′,7′-epoxybergamottin (purity ≥ 98%) were obtained from Cayman Chemical Company (Michigan, USA). Citropten (purity ≥ 99%) was purchased from Sigma-Aldrich (St. Louis, MO, USA). LCMS-grade methanol, LCMS-grade water, and HPLC-formic acid were purchased from Sigma-Aldrich (St. Louis, MO, USA). Stock solutions of each standard at a concentration of 10 ppm were prepared by diluting the powder in methanol.

### 3.2. Essential Oil Samples

*Citrus* volatile oils from trusted suppliers were obtained from the collection of the Aromatic Plant Research Center (APRC, Lehi, UT, USA). A total of 374 cold-pressed *Citrus* oil samples from the APRC collection are listed in Table 4. A simple dilute and shoot technique (1 μL oil in 999 μL of methanol) was used for sample preparation. Further dilution was performed whenever needed.

### 3.3. UPLC-MS/MS Analyses

Coumarins were quantified using a NEXERA UPLC system (Shimadzu Corp., Kyoto, Japan) equipped with a mass spectrometer (Triple quadrupole, LCMS8060, Shimadzu, Kyoto, Japan). Target compounds were chromatographed on a Shimadzu Nexcol C_18_ column (1.8 µm, 50 × 2.1 mm) with a C_18_ guard column (Restek, Bellefonte, PA, USA) at 40 °C. The mobile phase consisted of 0.1% formic acid in water (A) and 0.1% formic acid in methanol (B). The compounds were eluted using the following gradient: %10 B at 0 min, %20 B at 0.74 min, %60 B at 5.88 min, %90 B at 10 min, held at %100 B for 4 min, and %10 for 4 min before the next injection. The flow rate was maintained at 0.2 mL/min, and the injection volume was 1 μL. The UPLC system was connected to the MS by electrospray ionization (ESI) operating in positive ion mode. The interface, desolvation line, and heating block temperatures were 350, 250, and 400 °C, respectively. The capillary voltage was 4.5 kV, and CID gas was set at 350 kPa. Nebulizing gas flow was set at 3.0 L/min, and heating and drying gas were set at 10.0 L/min. The detection was completed in multiple reaction monitoring mode (MRM) (Table 5). Samples were run in triplicates with external standards in between. Each run contained a quality control (QC) standard, and at least one QC standard was run at the beginning and the end of the run. The acquired chromatographic results were processed in LabSolutions Insight software version 3.2 (Shimadzu). For each compound, calibration curves (0.005, 0.001, 0.0025, 0.005, 0.01, 0.025, 0.05, and 0.1 ppm) were drawn by linking its peak area and its concentration.

### 3.4. Method Validation

Method validation was executed according to the USP<1225> Validation of compendial procedures [31] and ICH harmonized tripartite guideline validation of analytical procedures: text and methodology Q2(R1) [32]. Specificity, precision, accuracy, linearity, intermediate precision, and limit of quantification (LOQ) were determined using standard solutions. Distilled yuzu essential oil was used as a matrix (total coumarins < 0.001 ppm). To prove the specificity of the method, standard solution mixtures and at least three blanks were processed to demonstrate the absence of interferences with the elution of the analytes. Precision and repeatability were determined by injecting six sample preparations spiked to a final concentration of 0.04 ppm and then calculating the RSD% between injections which may reach 10% for each. For the intermediate precision, the repeatability experiment was repeated on a second day and performed by a second analyst with the acceptance criterion of RSD ≤ 10 for each compound and each analyst. To determine the recoveries (accuracy) of the target compounds, three individually prepared samples of yuzu oil were spiked with three concentrations of the standard (LOQ, 0.04, and 0.05 ppm in triplicates). Recoveries were calculated by comparing the absolute peak areas with a reference measurement which must be within 80–120% of the expected value. Five concentrations from 0.001 to 0.1 ppm were used to determine linearity and a coefficient of determination (r) higher than 0.98 was needed. The data obtained during the linearity, precision, and accuracy studies were used to assess the range of the method for the target compounds. The acceptable range was defined as the concentration interval over which linearity, precision, and accuracy are acceptable. To estimate the LOQ, standard mixtures at low concentrations (0.0005 to 0.01 ppm) were analyzed. The calculated LOQ was determined using the signal-to-noise (S/N) ratio (10:1) and then injected 6 times. The acceptance criterion for the LOQ was RSD ≤ 15%. A calibration curve based on the linear range was prepared and injected to estimate the quantity of coumarins in the oil samples. Additionally, QC standards at low (0.05 ppm) and high (0.1 ppm) concentrations were used.

### 3.5. Multivariate Analysis

The average coumarin concentrations (12 compounds) in the *Citrus* samples were used as variables in the multivariate analysis. First, the data matrix was standardized by subtracting the mean for each compound concentration and dividing it by the standard deviation. For the agglomerative hierarchical cluster (AHC) analysis, the 24 *Citrus* samples were treated as operational taxonomic units (OTUs). Pearson correlation was selected as a measure of similarity, and the unweighted pair group method with arithmetic average (UPGMA) was used for cluster definition. Principal component analysis (PCA) was performed for the visual comparison of the coumarin compositions of the different *Citrus* groups using the 12 coumarin components as variables, with a Pearson correlation matrix. The AHC and PCA analyses were performed using XLSTAT v. 2018.1.1.62926 (Addinsoft, Paris, France).

## 4. Conclusions

In this study, we developed and validated a simple and sensitive UPLC-MS/MS method for the detection and quantification of 14 selected oxygen heterocyclic compounds (coumarins and furanocoumarins). Targeted screening using this method was successfully completed for the essential oils of 12 different *Citrus* species. To our knowledge, this is the most comprehensive investigation of coumarin and furanocoumarin profiles across commercial-scale *Citrus* oils to date. The lowest amount was detected in calamansi oil. Expressed oil of Italian bergamot showed the highest furanocoumarin content and the highest level of any individual furanocoumarin (bergamottin). Remarkable differences were observed in the coumarin and furanocoumarin levels among oils of different crop varieties and origins within the same species. We found potential correlations between bergapten and xanthotoxin which matches with known biosynthetic pathways. Patterns in furanocoumarin profiles lined up with known variations among the *Citrus* ancestral taxa. Using multivariate analysis, we were able to divide the *Citrus* oils into 5 main groups (bergamots; lime and German lemon; yuzu and grapefruit; oranges, tangerines, clementines, mandarins, calamansi, and petitgrains; and lemons) and correlate them to the coumarin compositions.

## Figures and Tables

**Table 1:** Linearity of the UPLC-MS Method (Equation and Coefficient of Determination, r^2^), Limit of Quantitation (LOQ), and Accuracy of the UPLC-MS Method of 14 Coumarins and Furanocoumarins.

Compound	Linearity			LOQ (ppm)	Accuracy	Precision	Intermediate Precision
	Linear Range (ppm)	Equation	r^2^		Recovery %	RSD%	RSD%
**Coumarins**							
Citropten	0.001–0.1	Y = 0.9847x + 0.0012	0.9991	0.001	98.92–113.80	2.60	4.44
5-Geranyloxy-7-methoxycoumarin	0.0001–0.1	Y = 0.9976x + 0.0002	0.9989	0.0001	94.07–105.44	2.23	2.05
Toncarine	0.005–0.1	Y = 0.9961x + 0.0003	0.9991	0.005	97.50–114.53	1.28	2.44
Herniarin	0.001–0.1	Y = 0.989x + 0.0008	0.9996	0.001	98.25–113.40	2.33	4.90
**Linear furanocoumarins**							
6’,7’-Epoxybergamottin	0.001–0.1	Y = 0.9948x + 0.0004	0.9990	0.001	96.83–109.00	2.29	1.05
Bergamottin	0.001–0.1	Y = 0.9975x + 0.0002	0.9988	0.001	95.75–104.56	2.26	3.05
Bergapten	0.0001–0.1	Y = 0.9956x + 0.0003	0.9994	0.0001	96.92–109.33	2.50	0.46
Biacangelicol	0.001–0.1	Y = 0.9923x + 0.0006	0.9992	0.001	97.17–107.27	2.18	2.32
Imperatorin	0.001–0.1	Y = 0.9904x + 0.0007	0.9997	0.001	96.40–106.67	2.59	2.32
Isopimpinellin	0.0001–0.1	Y = 1.0002x + 8x10-6	0.9989	0.0001	95.75–112.13	2.89	2.26
Oxypeucedanin	0.005–0.1	Y = 0.9946x + 0.0004	0.9987	0.005	96.33–107.73	2.62	1.17
Psoralen	0.001–0.1	Y = 0.9936x + 0.0005	0.9985	0.001	97.00–113.00	2.38	2.94
Trioxsalen	0.001–0.1	Y = 0.993x + 0.0005	0.9997	0.001	98.50–106.11	2.18	2.06
Xanthotoxin	0.001–0.1	Y = 0.9874x + 0.001	0.9997	0.001	98.58–112.07	3.67	2.54

**Table 2:** Total coumarin, total furanocoumarins, and coumarin distribution of the tested *Citrus* oils.

Citrus Oil	Total Coumarin (ppm)	Total FC (ppm)	Coumarin Distribution
Bergamot (Brazil)	52,473.90 ± 1775.63	48,798.90 ± 174.98	Bergamottin > imperatorin > bergapten > 5-geranyloxy-7-methoxycoumarin > citropten > xanthotoxin > 6’,7’-epoxybergamottin > herniarin > psoralen > oxypeucedanin > isopimpinellin > biacangelicol
Bergamot (Italy)	171,453.11 ± 9227.11	167,281.60 ± 1017.74	Bergamottin > imperatorin > 6’,7’-epoxybergamottin > 5-geranyloxy-7-methoxycoumarin > citropten > xanthotoxin > bergapten > herniarin > oxypeucedanin > isopimpinellin > psoralen > biacangelicol
Bitter Orange	814.95 ± 9.52	809.21 ± 1.30	6’,7’-Epoxybergamottin > xanthotoxin > bergapten > imperatorin > bergamottin > citropten > psoralen > 5-geranyloxy-7-methoxycoumarin > herniarin > isopimpinellin
Calamansi	0.15 ± 0.02	0	5-Geranyloxy-7-methoxycoumarin
Clementine (Brazil)	75.26 ± 0.08	43.03 ± 0.11	5-Geranyloxy-7-methoxycoumarin > bergamottin > imperatorin > citropten > oxypeucedanin > xanthotoxin > bergapten > herniarin > isopimpinellin
Clementine (Italy)	4.69 ± 0.03	3.38 ± 0.04	Oxypeucedanin > bergamottin > citropten > xanthotoxin > biacangelicol > bergapten > 5-geranyloxy-7-methoxycoumarin > psoralen
Grapefruit (Red)	13,099.29 ± 207.97	13,013.55 ± 22.87	6’,7’-Epoxybergamottin > bergamottin > imperatorin > oxypeucedanin > biacangelicol > xanthotoxin > bergapten > 5-geranyloxy-7-methoxycoumarin > citropten > isopimpinellin > psoralen > herniarin
Grapefruit (White)	9163.08 ± 229.85	9027.29 ± 25.14	6’,7’-Epoxybergamottin > imperatorin > bergamottin > oxypeucedanin > biacangelicol > xanthotoxin > psoralen > bergapten > herniarin > 5-geranyloxy-7-methoxycoumarin > isopimpinellin > citropten
Kaffir Lime	75.46 ± 5.13	43.15 ± 0.38	Imperatorin > citropten > bergamottin > 5-geranyloxy-7-methoxycoumarin > xanthotoxin > bergapten > oxypeucedanin > herniarin > isopimpinellin > psoralen
Kumquat	169.65 ± 0.72	93.19 ± 0.53	Bergamottin > 5-geranyloxy-7-methoxycoumarin > imperatorin > citropten > xanthotoxin > bergapten > oxypeucedanin > herniarin > isopimpinellin > 6′,7′-epoxybergamottin > biacangelicol
Lemon (Argentina)	5404.76 ± 3.60	3861.29 ± 3.41	Imperatorin > bergamottin > citropten > oxypeucedanin > 5-geranyloxy-7-methoxycoumarin > biacangelicol > 6′,7′-epoxybergamottin > herniarin > xanthotoxin > bergapten > isopimpinellin
Lemon (Brazil)	3321.86 ± 1.84	2335.29 ± 1.77	Imperatorin > bergamottin > citropten > 5-geranyloxy-7-methoxycoumarin > oxypeucedanin> biacangelicol > 6′,7′-epoxybergamottin > xanthotoxin > bergapten > herniarin > isopimpinellin
Lemon (Germany)	3107.99 ± 3.27	2029.93 ± 2.61	Imperatorin > bergamottin > 5-geranyloxy-7-methoxycoumarin > citropten > oxypeucedanin > 6′,7′-epoxybergamottin > biacangelicol > herniarin > xanthotoxin > bergapten > isopimpinellin
Lemon (Italy)	10,874.88 ± 8.28	8346.28 ± 9.30	Bergamottin > imperatorin > oxypeucedanin > 5-geranyloxy-7-methoxycoumarin > citropten > biacangelicol > 6′,7′-epoxybergamottin > xanthotoxin > bergapten > herniarin > isopimpinellin > psoralen
Lemon (South Africa)	4268.48 ± 2.13	3185.78 ± 2.81	Oxypeucedanin > imperatorin > bergamottin > citropten > 5-geranyloxy-7-methoxycoumarin > biacangelicol > 6′,7′-epoxybergamottin > xanthotoxin > bergapten > herniarin > isopimpinellin
Lemon (Spain)	3343.46 ± 4.76	2467.31 ± 4.28	Imperatorin > bergamottin > 5-geranyloxy-7-methoxycoumarin > citropten > oxypeucedanin > 6′,7′-epoxybergamottin > biacangelicol > xanthotoxin > bergapten > herniarin > isopimpinellin
Lemon (USA)	2717.40 ± 4.45	1985.52 ± 4.93	Imperatorin > bergamottin > citropten > 5-geranyloxy-7-methoxycoumarin > oxypeucedanin > biacangelicol > 6′,7′-epoxybergamottin > xanthotoxin > bergapten > herniarin > isopimpinellin > psoralen
Lime	23,795.43 ± 564.22	16,725.07 ± 43.80	Bergamottin > imperatorin > 5-geranyloxy-7-methoxycoumarin > citropten > oxypeucedanin > xanthotoxin > herniarin > bergapten > isopimpinellin > 6′,7′-epoxybergamottin > biacangelicol > psoralen
Mandarin (Green)	32.27 ± 0.35	22.77 ± 0.46	Imperatorin > bergamottin > 5-geranyloxy-7-methoxycoumarin > citropten > xanthotoxin > oxypeucedanin > herniarin > bergapten > isopimpinellin > biacangelicol
Mandarin (Red)	27.42 ± 0.06	19.06 ± 0.08	Imperatorin > bergamottin > 5-geranyloxy-7-methoxycoumarin > citropten > oxypeucedanin > xanthotoxin > bergapten > herniarin > isopimpinellin > biacangelicol
Mandarin (Yellow)	52.89 ± 0.04	37.96 ± 0.05	Imperatorin > bergamottin > 5-geranyloxy-7-methoxycoumarin > citropten > xanthotoxin > bergapten > herniarin > oxypeucedanin > isopimpinellin > biacangelicol
Petitgrain (Lemon)	36.22 ± 2.10	20.74 ± 0.18	Herniarin > imperatorin > citropten > bergapten > xanthotoxin > bergamottin > psoralen > 5-geranyloxy-7-methoxycoumarin > oxypeucedanin > isopimpinellin > biacangelicol
Petitgrain (Lime)	58.93 ± 1.37	47.47 ± 0.11	Imperatorin > xanthotoxin > citropten > bergapten > isopimpinellin > bergamottin > 5-geranyloxy-7-methoxycoumarin > herniarin > oxypeucedanin > psoralen
Sweet Orange (Navel)	179.26 ± 9.94	140.13 ± 0.75	6′,7′-Epoxybergamottin > bergamottin > oxypeucedanin > imperatorin > 5-geranyloxy-7-methoxycoumarin > citropten > biacangelicol > xanthotoxin > bergapten > isopimpinellin > herniarin > psoralen
Sweet Orange (Valencia)	122.27 ± 2.29	68.75 ± 0.19	5-Geranyloxy-7-methoxycoumarin > imperatorin > bergamottin > citropten > oxypeucedanin > xanthotoxin > bergapten > herniarin > isopimpinellin > 6′,7′-epoxybergamottin > biacangelicol
Tangerine (Brazil)	149.45 ± 1.58	94.31 ± 1.45	Imperatorin > bergamottin > 5-geranyloxy-7-methoxycoumarin > citropten > xanthotoxin > bergapten > 6′,7′-epoxybergamottin > oxypeucedanin > herniarin > isopimpinellin > biacangelicol > psoralen
Tangerine (Italy)	2.44 ± 0.02	1.95 ± 0.03	Bergamottin > bergapten > xanthotoxin > oxypeucedanin > citropten > 6′,7′-epoxybergamottin > 5-geranyloxy-7-methoxycoumarin
Yuzu	609.06 ± 0.33	597.1 ± 0.41	6′,7′-Epoxybegamottin > biacangelicol > oxypeucedanin > xanthotoxin > bergapten > imperatorin > citropten > bergamottin > herniarin > 5-Geranyloxy-7-methoxycoumarin > isopimpinellin

**Table 3:** Non-volatile components of cold-pressed *Citrus* oils that are reported in the literature.

Citrus Oil	Non-Volatile Components	Reported Amount	Reference(s)
**Bitter orange**	Bergapten Epoxybergamottin Psoralen	0.035–0.073% 0.082% 0.007%	[3]
**Bergamot CP**	5-Geranloxy-7-methoxycoumarin 5-Methoxy-7-geranoxycoumarin Bergamottin Bergaptol Psoralen Bergapten Citropten	0.08–0.68% 0.04–0.15% 0.68–2.75% 0–0.19% 0–0.0026% 0.11–0.33% 0.01–0.35%	[3]
**Lemon**	5-Geranloxy-7-methoxycoumarin 8-Geranyloxypsoralen Bergamottin Byakangelicol Bergapten Citropten Isopimpinellin oxypeucedanin	0.18–0.28% 0.01–0.045% 0.16–0.54% 0.006–0.16% 0.0001–0.035% 0.05–0.17% 0–0.011% 0.09–0.82%	[3]
**Lime**	5-Geranloxy-7-methoxycoumarin 5-Geranoxy-8-methoxypsoralen 8-Geranyloxypsoralen 5-Methoxy-7-geranoxycoumarin Bergamottin Bergapten Citropten Isopimpinellin oxypeucedanin	1.7–3.2% 0.2–0.9% 0.10–0.14% 1.7–5.2% 1.7–3.0% 0.17–0.33% 0.4–2.2% 0.1–1.3% 0.02–0.3%	[3]
**Grapefruit**	Bergamottin Epoxybergamottin Bergapten	<0.11% 0.1126% 0.012–0.19%	[3]
**Mandarin**	Bergamottin Bergapten	0–0.001% 0–0.0003%	[3]
**Mandarin CO_2_**	Bergapten Citropten	0.07% 0.76%	[20]
**Lemon (coastal)**	Bergapten Citropten Herniarin Isopimpinellin	0–10 ppm 700–1300 ppm 0–10 ppm 0–5 ppm	[21]
**Lemon (desert)**	Bergapten Citropten Herniarin Isopimpinellin	50–350 ppm 700–1700 ppm <10 ppm 35–110 ppm	[21]
**Lemon**	5-Geranoxy-7-methoxycoumarin 5-Isopent-2′-enyloxy-8-(2′,3′-epoxyisopentyloxypsoralen) 5-Isopentenyloxy-7-methoxycoumarin 8-Geranyloxypsoralen Bergamottin Byakangelicol Citropten Isoimperatorin Oxypeucedanin Oxypeucedanin hydrate	1800–2500 ppm 190–370 ppm tr 190–360 ppm 1600–1910 ppm 660–1230 ppm 520–1420 ppm tr 890–1570 ppm tr	[21]
**Lemon**	5-Geranoxy-7-methoxycoumarin 5-Isopent-2′-enyloxy-8-(2′,3′-epoxyisopentyloxypsoralen) 8-Geranyloxypsoralen Bergamottin Byakangelicol Citropten Oxypeucedanin	2453–2845 ppm 204–324 ppm 399–454 ppm 2635–2973 ppm 555–1640 ppm 659–1495 ppm 863–2200 ppm	[22]
**Lime oil (Mexican type B)**	5-Geranoxy-7-methoxycoumarin 5-Isopentenyloxy-7-methoxycoumarin 8-Geranyloxypsoralen Bergamottin Bergapten Byakangelicol Citropten Cnidicin Herniarin Imperatorin Isoimperatorin Isopimpinellin Oxypeucedanin Oxypeucedanin hydrate	27,770–45,350 ppm 2100–2790 ppm 3800–4540 ppm 25,320–41,590 ppm 2160–3920 ppm 80–1020 ppm 5940–10,950 ppm 70–250 ppm 3350–4670 ppm 380–660 ppm 70–410 ppm 3010–7300 ppm 6660–10,720 ppm 1620–1710 ppm	[23]
**Lime (type A)**	5-Geranoxy-7-methoxycoumarin 5-Isopentenyloxy-7-methoxycoumarin 8-Geranyloxypsoralen Bergamottin Bergapten Byakangelicol Citropten Cnidicin Herniarin Imperatorin Isopimpinellin Oxypeucedanin Oxypeucedanin hydrate	41,550–63,320 ppm 4170–4830 ppm 6520–8100 ppm 37,300–56,130 ppm 2000–3450 ppm 0–90 ppm 7350–11,740 ppm 90–340 ppm 1460–2970 ppm 830–900 ppm 5670–10,210 ppm 0–260 ppm 780–1160 ppm	[23]
**Key lime CP**	5-Isopentenyloxy-7-methoxycoumarin 8-Geranyloxypsoralen Bergamottin Bergapten Byakangelicol Citropten Cnidicin Cnidilin Herniarin Imperatorin Isoimperatorin Isopimpinellin Oxypeucedanin Oxypeucedanin hydrate	2790 ± 15 ppm 4470 ± 28.7 ppm 36,401 ± 150.9 ppm 3000 ± 31.1 ppm 92 ± 9.9 ppm 10,950 ± 92.8 ppm 250 ± 62 ppm 249 ± 7.6 ppm 3880 ± 45.8 ppm 39 ± 10.3 ppm 88 ± 5.9 ppm 7300 ± 46.9 ppm 10,600 ± 85.1 ppm 1690 ± 203 ppm	[24]
**Key Lime (type A)**	5-Geranoxy-7-methoxycoumarin 5-Isopentenyloxy-7-methoxycoumarin 8-Geranyloxypsoralen Bergamottin Bergapten Citropten Cnidilin Herniarin Isoimperatorin Isopimpinellin Oxypeucedanin hydrate Xanthotoxin	306.5–404.5 ppm <0.1 ppm <0.1 ppm 315.7–328.3 ppm 10–12.4 ppm 49.1–63.2 ppm 2.5–3.5 ppm 8.6–9.6 ppm <0.1 ppm 35–36.5 ppm <0.1 ppm <0.1 ppm	[25]
**Key Lime (type A)**	5-Geranoxy-7-methoxycoumarin 5-Isopentenyloxy-7-methoxycoumarin 8-Geranyloxypsoralen Bergamottin Bergapten Citropten Cnidilin Herniarin Isoimperatorin Isopimpinellin Oxypeucedanin Oxypeucedanin hydrate Xanthotoxin	409.3 ppm <0.1 ppm <0.1 ppm 315.4 ppm 8.9 ppm 48.4 ppm 2.4 ppm 7.4 ppm <0.1 ppm 33.1 ppm 14.4 ppm <0.1 ppm <0.1 ppm	[25]
**Persian Lime**	5-Geranoxy-7-methoxycoumarin 5-Isopentenyloxy-7-methoxycoumarin 8-Geranyloxypsoralen Bergamottin Bergapten Citropten Cnidilin Herniarin Isoimperatorin Isopimpinellin Oxypeucedanin Oxypeucedanin hydrate Xanthotoxin	194.3–378 ppm <0.1 ppm <0.1 ppm 222.1–391.8 ppm 15.8–25 ppm 32.6–56.9 ppm 0.5–0.8 ppm 33.9–59.4 ppm <0.1 ppm 16.9–29.3 ppm 21–32.8 ppm <0.1 ppm <0.1 ppm	[25]
**Bergamot oil (Italian)**	5-Geranoxy-7-methoxycoumarin Bergamottin Bergapten Citropten	0.14–0.18% 1.37–1.6% 0.18–0.21% 0.18–0.26%	[25]
**Bergamot CP**	5-Geranoxy-7-methoxycoumarin Bergamottin Bergapten Citropten	8–27 ppm 100–275 ppm 10–32 ppm 12–35 ppm	[25]
**Bergamot oil (commercial)**	5-Geranoxy-7-methoxycoumarin 5-Geranyloxy-8-methoxypsoralen 5-Isopentenyl-8-(2′,3′-dihydroxyisopentyloxy)psoralen 5-Isopentenyloxy-7-methoxycoumarin 8-Geranyloxypsoralen Bergamottin Bergapten Citropten Herniarin Isopimpinellin Oxypeucedanin	18–37 ppm <5 ppm <5 ppm <5 ppm <5 ppm 68–116 ppm 4–10 ppm 10–13 ppm <5 ppm <5 ppm <5 ppm	[25]
**Bergamot oil**	Bergamottin Bergapten Citropten	96.7 ug/100mg 152.5 ug/100mg 21.7 ug/100mg	[26]
**Bergamot**	Bergamottin Bergapten Epoxybergamottin Oxypeucedanin	16,312 ppm 8 ppm 70.3 ppm 53.5 ppm	[12]
**Bergamot**	5-Geranoxy-7-methoxycoumarin Bergamottin Bergapten Citropten	0.08–0.104% 1.097–1.409% 0.138–0.209% 0.134–0.212%	[27]
**Bergamot**	5-Geranoxy-7-methoxycoumarin Bergamottin Bergapten Citropten Herniarin	0–2.827 ppm 0–39.203 ppm 0–4.215 ppm 0–6.134 ppm 0–0.251 ppm	[28]
**Bergamot**	Bergapten Citropten	1.70% 0.40%	[29]
**Bergamot**	5-Geranoxy-7-methoxycoumarin Bergamottin Bergapten Citropten	0–3 ppm 0–37 ppm 0–268 ppm 0–14 ppm	[30]
**Bergamot**	5-Geranoxy-7-methoxycoumarin Bergamottin Bergapten Citropten Herniarin	1065 ± 7.5 ppm 19,605 ± 73.2 ppm 2474 ± 28.4 ppm 2232 ± 26.3 ppm 67 ± 3.2 ppm	[24]
**Bergamot**	Bergamottin Bergapten Citropten	1.14–2.73% 0.06–0.4% 0.1–0.3%	[25]

**Table 4:** Sample information of citrus essential oil samples from the APRC collection.

*Citrus* Oil	Scientific Name	No. of Samples	Origin
Calamansi	*Citrus* × *microcarpa* (Bunge) Wijnands	5	Philippines
Tangerine	*Citrus tangerina* Hort. Ex Tanaka	13	Brazil
Kumquat	*Citrus japonica* Thunb	3	Brazil
Mandarin	*Citrus reticulata* Blanco	33	Brazil
Clementine	*Citrus clementina* Hort. Ex Tanaka	6	Brazil
Yuzu or Yuja	*Citrus junos* Sieb. Ex Tanaka	16	Brazil
Bitter Orange	*Citrus aurantium* L.	6	Japan
Sweet Orange	*Citrus sinensis* L.	36	Brazil
Lime	*Citrus aurantifolia* (Christm.) Swingle	28	Brazil
Bergamot	*Citrus bergamia* Risso & Poit	66	Italy and Brazil
Grapefruit	*Citrus* × *paradisi* Macfady	45	South Africa and USA
Lemon	*Citrus limon* Osbeck	97	Spain, Argentina, Brazil, Italy, USA, South Africa, and Germany
Petitgrain	*Citrus aurantifolia* leaf and *Citrus limon* leaf	20	Paraguay

**Table 5:** Multiple reaction monitoring mode (MRM) parameters.

Name	Other Name(s)	CAS #	Precursor (m/z)	Product 1 (m/z)	Product 2 (m/z)	Product 3 (m/z)	RT (min)
**Coumarins**							
Citropten or Limettin	5,7-dimethoxycoumarin	487-06-9	206.90	192.10	149.10	121.15	7.61
5-Geranyloxy-7-methoxycoumarin		7380-39-4	328.90	193.10	137.05	149.10	12.25
Herniarin	7-Methoxycoumarin	531-59-9	176.90	121.05	78.10	77.10	6.58
Toncarine	6-Methylcoumarin	92-48-8	160.90	105.05	76.95	115.05	7.18
**Linear furanocoumarins**							
Xanthotoxin	8-methoxypsoralen	298-81-7	216.90	89.05	174.10	202.10	7.74
Bergamottin	5-geranyloxypsoralen	7380-40-7	339.00	203.00	147.05	91.15	12.09
Oxypeucedanin	5-(2l,3l-epoxyisopentyloxy)psoralen	26091-73-6	286.90	202.90	147.20	91.20	8.34
Biacangelicol or Byakangelicol	5-methoxy-8-(2l,3l-epoxyisopentyloxy) psoralen	26091-79-2	317.00	233.05	231.10	218.10	8.21
Psoralen		66-97-7	186.90	131.10	77.10	115.10	6.98
Isopimpinellin		482-27-9	246.90	216.95	232.05	189.05	7.57
Bergapten		484-20-8	216.90	202.10	174.10	89.05	7.75
Imperatorin	8-isopentenyloxypsoralen	482-44-0	202.90	91.15	91.15	65.10	12.09
Trioxsalen		3902-71-4	229.00	115.15	142.20	128.10	9.62
6′,7′-Epoxybergamottin		206978-14-5	354.90	203.10	153.15	147.10	10.11

## Data Availability

All data are available upon reasonable request from the corresponding author (N.S.D.).

## Abbreviations

AHC	agglomerative hierarchical cluster analysis
DAD	diode array detector
ELISA	enzyme-linked immunosorbent assay
EO	essential oil
FC	furanocoumarin
GC-MS	gas chromatography–mass spectroscopy
HPLC	high-performance liquid chromatography
LC-MS	liquid chromatography- mass spectrometry
LOQ	limit of quantification
MRM	multiple reaction monitoring mode
NMR	nuclear magnetic resonance
OTUs	operational taxonomic units
PCA	Principal component analysis
ppm	parts per million
QC	quality control
RP-HPLC	reversed-phase-high-performance liquid chromatography
UPGMA	unweighted pair group method with arithmetic average
UPLC-MS/MS	ultra-performance liquid chromatography-tandem mass spectrometry
